# Chronic *Plasmodium brasilianum* infections in wild Peruvian tamarins

**DOI:** 10.1371/journal.pone.0184504

**Published:** 2017-09-13

**Authors:** Gideon A. Erkenswick, Mrinalini Watsa, M. Andreína Pacheco, Ananias A. Escalante, Patricia G. Parker

**Affiliations:** 1 Department of Biology and Whitney R. Harris World Ecology Center, University of Missouri-St. Louis, Saint Louis, Missouri, United States of America; 2 Field Projects International, Saint Louis, Missouri, United States of America; 3 Department of Anthropology, Washington University in St. Louis, Saint Louis, Missouri, United States of America; 4 Department of Biology/Institute for Genomics and Evolutionary Medicine (igem), Temple University, Philadelphia, Pennsylvania, United States of America; 5 WildCare Institute, Saint Louis Zoo, Saint Louis, Missouri, United States of America; Université Pierre et Marie Curie, FRANCE

## Abstract

There is an increased interest in potential zoonotic malarias. To date, *Plasmodium malariae* that infects humans remains indistinguishable from *Plasmodium brasilianum*, which is widespread among New World primates. Distributed throughout tropical Central and South America, the Callitrichidae are small arboreal primates in which detection of natural *Plasmodium* infection has been extremely rare. Most prior screening efforts have been limited to small samples, the use of low-probability detection methods, or both. Rarely have screening efforts implemented a longitudinal sampling design. Through an annual mark-recapture program of two sympatric callitrichids, the emperor (*Saguinus imperator*) and saddleback (*Saguinus fuscicollis*) tamarins, whole blood samples were screened for *Plasmodium* by microscopy and nested PCR of the cytochrome b gene across four consecutive years (2012–2015). Following the first field season, approximately 50% of the samples collected each subsequent year were from recaptured individuals. In particular, out of 245 samples from 129 individuals, 11 samples from 6 individuals were positive for *Plasmodium*, and all but one of these infections was found in *S*. *imperator*. Importantly, the cytochrome b sequences were 100% identical to former isolates of *P*. *malariae* from humans and *P*. *brasilianum* from *Saimiri* sp. Chronic infections were detected as evidenced by repeated infections (7) from two individuals across the 4-year study period. Furthermore, 4 of the 5 infected emperor tamarins were part of a single group spanning the entire study period. Overall, the low prevalence reported here is consistent with previous findings. This study identifies two new natural hosts for *P*. *brasilianum* and provides evidence in support of chronic infections in wildlife populations. Given that callitrichids are often found in mixed-species associations with other primates and can be resilient to human-disturbed environments, they could contribute to the maintenance of *P*. *malariae* populations if future work provides entomological and epidemiological evidence indicating human zoonotic infections.

## Introduction

In 2015 malaria was diagnosed in approximately 212 million people, and resulted in the loss of 438,000 lives worldwide [1]. In malaria-endemic regions, infections are unevenly distributed among human populations, with the highest prevalence among children and adolescents. Today, malaria control programs remain among the largest public health efforts, costing an estimated $4.75 billion annually [2], despite the fact that the causative agents of malaria (protozoan parasites of the genus *Plasmodium*) were first discovered as early as 1880 [3]. A potential challenge faced by ongoing efforts to eliminate malaria in human communities is the possibility of zoonotic infections [4]. In particular, there is compelling evidence that some *Plasmodium* species infecting humans are also circulating in nearby simian and ape communities. Whether such non-human primate host can act as a reservoir of human malarias is a matter of great interest.

According to the Global Mammal Parasite Database, 27 species of *Plasmodium* have been documented in nonhuman primates [5], three of which (*Plasmodium falciparum*, *Plasmodium vivax*, *Plasmodium knowlesi*, and *Plasmodium malariae*) frequently occur in humans. Along with *Plasmodium ovale*, *Plasmodium* species that infect humans do not form a monophyletic group [6]. The two parasites that cause the greatest morbidity (*P*. *falciparum* and *P*. *vivax*) are part of larger clades of species that include many that parasitize nonhuman primates [4,7,8].

In South America, *Plasmodium brasilianum* was first described in monkeys in the beginning of the 20^th^ century and has now been documented in approximately 31 species of New World monkeys [9,10]. This broad host range is unusual among other non-human primate malarias and may indicate a very resilient parasite species. To date, numerous studies have looked for, but not found, any reliable morphological, serological, or genetic differences between *P*. *brasilianum* and *Plasmodium malariae* that infect humans [7,11–14]. Lalremruata et al. [13] collected blood samples from several remote populations of the Yanomami people in Venezuela, and isolated 33 infections by nested-PCR screening for the small subunit ribosomal RNA gene (18S SSU rRNA) of *P*. *malariae*. Of these, 12 sequences were 100% identical to *P*. *brasilianum* strains recovered from howler monkeys in French Guiana, and the remainder were 99–100% identical to *P*. *malariae* strains from Myanmar and Papua New Guinea. Although the 18S gene does not allow us to accurately discern recent host switches due to its rate and semi-concerted mode of evolution [15], the genetic distances between all 33 strains seems consistent with intraspecific variation observed within other single species of *Plasmodium*. The only factor that has ever been used to differentiate these two parasites is host-identity (human or not), and yet experimental studies have demonstrated that nonhuman primates are susceptible to *P*. *malariae* [16]. These findings suggest that *P*. *brasilianum* and *P*. *malariae* could very well be the same organism.

*P*. *malariae/brasilianum* causes quartan malaria, so termed for having 72-hour erythrocytic cycles, unlike *P*. *falciparum* or *P*. *vivax* that cycle every 48 hours [17]. *P*. *malariae* has led to nephrotic syndromes in humans and experimentally infected monkeys [17,18], and has been shown to persist in humans for years, suggesting that a similar pattern may occur in non-human primates like chimpanzees [19,20]. Recrudescent infections of *P*. *malariae/brasilianum* can occur when hosts are subjected to stressful conditions or become immunocompromised [17,21]. In malaria-endemic regions, co-infections of *P*. *malariae/brasilianum* with other *Plasmodium* spp. are common [13,22,23]. Since host parasitemia for *P*. *malariae/brasilianum* is relatively low, co-infections are probably under-detected when screenings are performed only by microscopy [17,18], and yet microscopy remains the most broadly utilized diagnostic technique around the world today.

The ability to reside in non-human primates, induce renal pathology, persist as a chronic infection in humans, and interact with other species of *Plasmodium* qualifies *P*. *malariae* as an important health concern at the human-wildlife interface given that present research suggests it is the same as *P*. *brasilianum*. Fundamental to assessing health risks are the development of a clear understanding of host breadth and associated prevalence for these (or this) species. The majority of infections found in wild New World monkeys are confined to the Atelidae, Pitheciidae, and Cebidae, while detection in the Callitrichidae, perhaps the most speciose and widespread Neotropical primate family, remains rare [9,10,14,22]. Callitrichids are always found in sympatry with other New World primate species, frequently in the context of mixed-species, or polyspecific associations [24,25]. Unlike the majority of other New World primates they can also persist in disturbed and human occupied areas [26–28]. Moreover, continuing removal of large Neotropical primates due to poaching and hunting may be leading to a population expansion of the callitrichids [29]. If proven to be reservoirs of *Plasmodium* infections, these characteristics of the callitrichids might implicate them as an important sylvatic component for malaria control efforts.

When considering accessible survey data on *Plasmodium* infections from wild populations of callitrichids to date, several biases stand out. First, although five of the seven callitrichid genera have been tested for *P*. *brasilianum* (*Saguinus*, *Cebuella*, *Callithrix*, *Leontopithecus*, *and Mico*), the majority of species have not been sampled [5,10]. Of the ~24 species investigated so far, nine have shown an infection, including *Saguinus midas*, *Saguinus niger*, *Saguinus geoffroyi*, *Saguinus martinsi*, *Mico humeralifer*, *Leontopithicus chrysomelas*, *Leontopithecus chrysopygus*, *Leontopithecus*. *rosalia*, and *Callithrix geoffroyi*. However, only the first three of these species (*S*. *midas*, *S*. *niger*, and *S*. *geoffroyi*) are clear cases of infection in natural environments; the other infections were recently detected from captive animals at primate research or rescue centers [10,30]. Of the cases representing natural infections, the number of infected individuals and corresponding sample sizes consist of 1/1000 for *S*. *geoffroyi* [31], 4/109 for *S*. *niger* [32], 4/178 in another study of *S*. *niger* [32,33], and 3 in 54 for *S*. *midas* [14]. This is relevant since *P*. *brasilianum* tends to exhibit low prevalence, averaging 0.045±0.043 for the Callitrichidae and 0.023±0.024 for the Primate Order[5]. Third, no studies to date report chronic natural infections by sampling the same individuals across years. If true, this would provide evidence that the Callitrichidae could be suitable hosts for *P*. *malariae/brasilianum* and may act as a reservoir for human malaria.

Here we screen for natural *Plasmodium* infections longitudinally across four years in two sympatric species of callitrichids, the saddleback tamarin, *Saguinus fuscicollis weddelli* (but see ongoing taxonomic revisions [34,35]), and the emperor tamarin, *Saguinus imperator*. Given the observed host breadth of *P*. *brasilianum/malariae* and the small sample sizes of prior efforts, we predict that callitrichine species could be competent reservoirs for this parasite, in which *Plasmodium* is permanently maintained and infections are vectored between conspecifics [36]. Second, since *P*. *malariae* infection can be chronic in humans, we predict that the same will be true for these callitrichine hosts. In addition to testing these predictions, our goal is to establish the prevalence of *Plasmodium*, and incidence of new infections, in both species, and to explore any patterns in how infections are distributed across host characteristics such as sex, age class, and group membership.

## Materials and methods

### Study subjects and sampling

**Fig 1:**
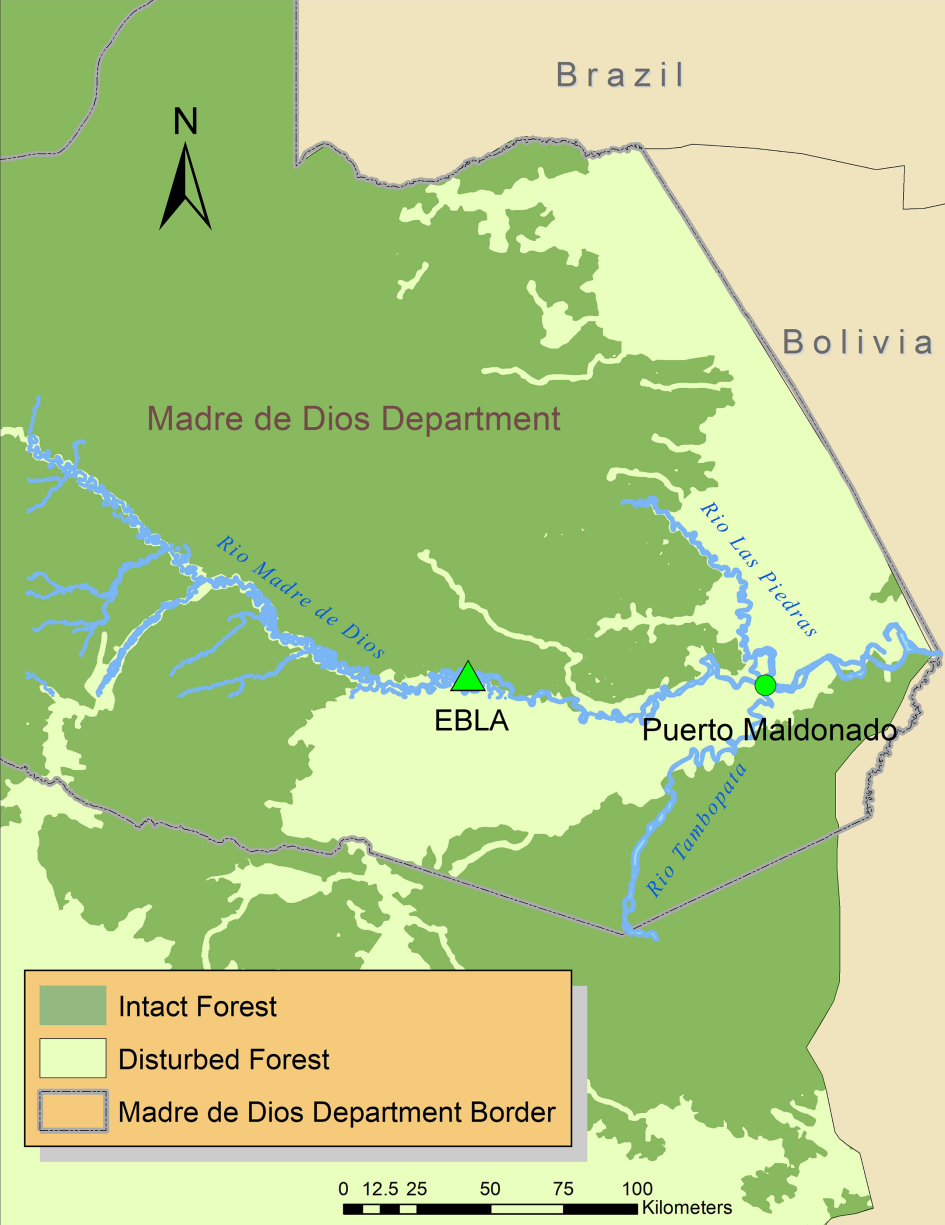
Field site and surrounding area. Spatial data layers on intact forest coverage were obtained from the Intact Forest Landscapes project [38].

Samples were collected from a free-ranging population of saddleback (*Saguinus fuscicollis*) and emperor (*Saguinus imperator*) tamarins at the Estación Biológica Rio Los Amigos (EBLA) in the Madre de Dios Department of southeastern Perú (12°34’07”S, 70°05’57”W) (Fig 1). This privately owned field station is managed and protected by the Amazon Conservation Association (ACA) and receives more than 150 visitors each year. The field station is located at the confluence of the Los Amigos and Madre de Dios Rivers, approximately 99 kilometers east of Puerto Maldonado, the state capital. All of the Madre de Dios Department is identified as a human malaria transmission zone [37]. The 900-ha field station is contiguous with the much larger Los Amigos Conservation Concession that lies within the buffer zone of Manu National Park. The site exhibits lower densities of large-bodied primates than has been recorded from nearby forest in the government protected Tambopata National Reserve, which is attributed to hunting that took place prior to purchase by the ACA [29]; however densities of medium- and small-bodied primates are higher. The study groups of both species inhabit both *terra firme* and *várzea* habitat.

Since 2009, we have encountered approximately 70 unique individuals each year, across both species. Our program is optimized to ensure habituation of primates to subsequent human observation [39]. Animals are given permanent identification tags via subcutaneous microchips (Avid, Home Again©) so samples could be collected from the same individual across years. Samples for this study were collected across four years (2012–2015) in June and July (the dry season). During capture, blood samples of < 300 uL were drawn from the femoral vein of each animal while it was anesthetized with ketamine hydrochloride (Ketalar, Pfizer Inc., New York, USA). Each sample was stored dry on Whatman FTA Micro Elute Cards for subsequent DNA extraction and at least two blood smears were prepared with fresh blood. All sampling protocols adhere to guidelines outlined by the American Society of Mammalogists [40] and were approved by the Institutional Animal Care and Use Committee at the University of Missouri-St. Louis (317006–2, 733363–2) and the Directorate of Forest and Wildlife Management (DGFFS) of Perú annually.

### Blood parasite microscopy

Immediately after blood draw, blood smears were made on standard microscope slides and air-dried. All smears were fixed for five minutes in 100% methanol within six hours and stained in Giemsa’s solution following Valkiunas et al. [41] within three weeks of fixation. Smears were observed at 400x magnification using light microscopy (Olympus CX31) for the presence of parasites. Blood parasites were recorded while conducting a total leukocyte count estimation (enumeration of leukocytes in 10 non-overlapping fields of view in the smears’ monolayer at 400x magnification) and differential (classification of 200 leukocytes in the monolayer at 1000x magnification); each slide examination took approximately 25 minutes. Examinations were carried out in a systematic direction to avoid overlapping fields of view, excluding damaged sections, where leukocytes and parasites were too distorted to identify.

### Molecular detection and sequencing

DNA was isolated from a 3 mm diameter hole punch from the blood stored on Whatman FTA Micro Elute Cards into 30ul of ddH_2_0 using standardized protocols recommended by the manufacturer (GE Health Care Life Sciences, Pittsburgh USA). DNA samples from the first three years (2012–2014) were screened for haemosporidian parasites by a nested polymerase chain reaction (nPCR) protocol that targets part of the parasite cytochrome b (*cytb*) gene, 709 base pairs (bp), following Duval et al. [42]. To confirm infection status and to obtain the near complete mitochondrial *cytb* gene (1,131bp) for infected individuals across the entire study period (2012–2015), we employed a separate nPCR protocol that amplifies a 1,038 bp fragment with specific forward-TGTAATGCCTAGACGTATTCC and reverse-GTCAAWCAAACATGAATATAGAC primers for the outer PCR and forward-TCTATTAATTTAGYWAAAGCAC and reverse-GCTTGGGAGCTGTAATCATAAT primers for the inner PCR, following Pacheco et al. [43]. PCR amplifications were carried out in a 50 μl volume with 8ul of total genomic DNA, 2.5 mM MgCl_2_, 1X PCR buffer, 1.25 mM of each deoxynucleoside triphosphate, 0.4 mM of each primer, and 0.03 U/μl AmpliTaq polymerase (Applied Biosystems, Roche-USA). The PCR conditions were: a partial denaturation at 94°C for 4 min, 36 cycles of 1 min at 94°C, 1 min at 53°C and 2 min extension at 72°C, and a final extension of 10 min added to the last cycle. Then, a nested PCR using 1 μl of the first amplification as the template was performed under identical PCR conditions. After electrophoresis, all amplified products were excised from the gels, purified by the QIAmp Gel Extraction Kit (Qiagen), and both strands were sequenced using an Applied Biosystems 3730 capillary sequencer.

### Phylogenetic analysis

Complete *cytb* gene sequence identity for samples positive for *Plasmodium* was confirmed using BLAST against NCBI. Electropherograms were visually examined to rule out mixed infections. In addition to the sequences obtained in this study, we included a total of 26 sequences available in GenBank for *Plasmodium* parasites isolated from mammals in the subsequent phylogenetic analysis. The phylogenetic relationships between sequences were inferred on the *cytb* gene using MrBayes v3.2.6 with the default priors [44]. Alignments were made using ClustalX v2.0.12 and Muscle as implemented in SeaView v4.3.5 [45] with manual editing. The data were fit with a General Time-Reversible model (GTR + I + Γ) that had the lowest Bayesian Information Criterion (BIC) score [46]. Bayesian support for the nodes was inferred in MrBayes using 4 × 10^6^ Markov Chain Monte Carlo (MCMC) steps, and after convergence was reached (posterior probability < 0.01, potential scale reduction factor between 1.00 and 1.02), we discarded 25% of the samples as burn-in [44]. Then, the sequence divergence between species was calculated using a Kimura two-parameter model of substitution as implemented in MEGA v.6.05 [47].

## Results

**Fig 2:**
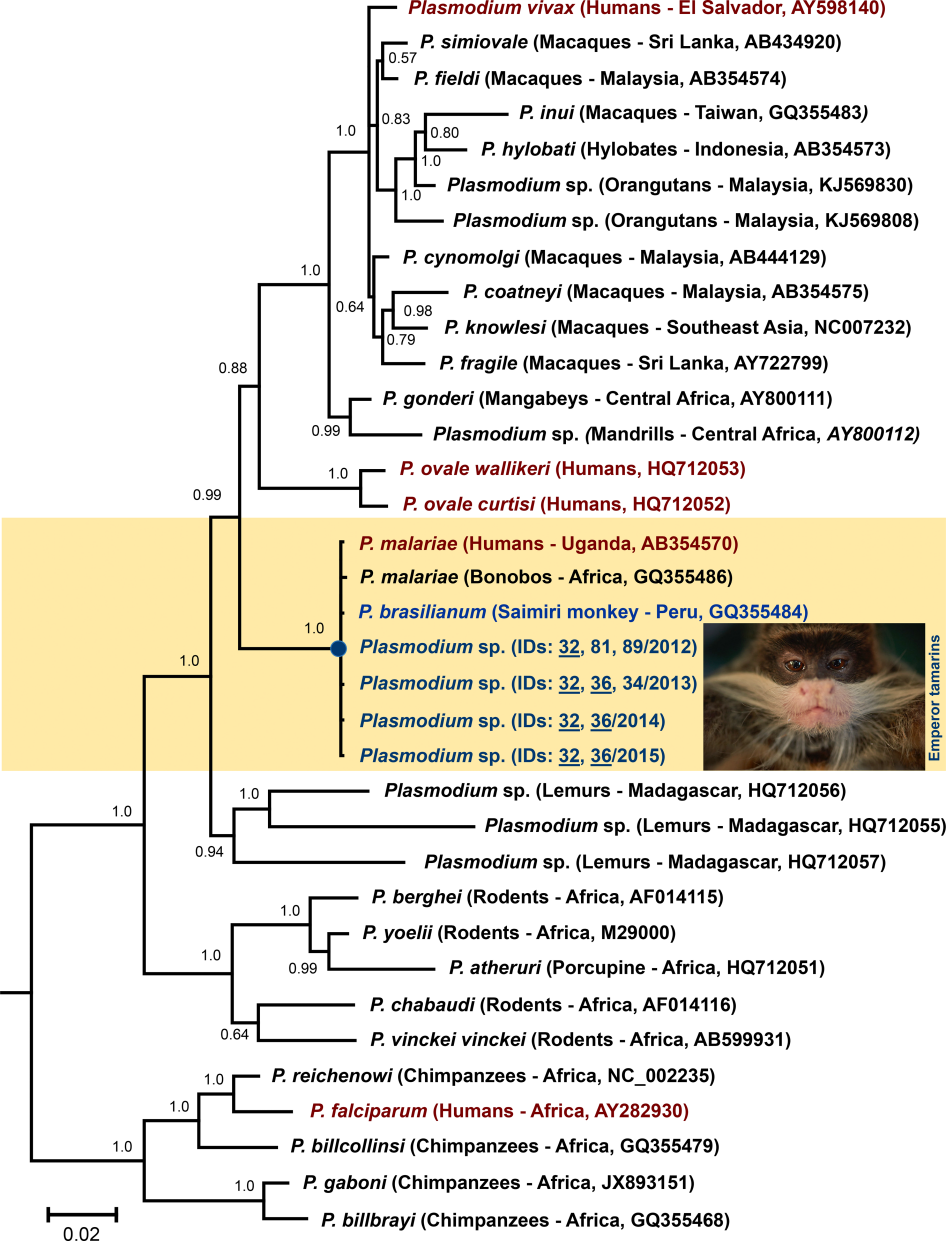
Cytochrome b phylogeny with new *Plasmodium* isolates. One *Plasmodium* isolate per year from this study has been included in the phylogeny with infected animals indicated by their unique animal ID numbers. *Plasmodium* isolates from humans are indicated in red and a squirrel monkey isolate from Perú is in blue. For each sequence, host species, sample locations, and GenBank accession numbers are provided. Emperor tamarin photo reprinted from https://fieldprojects.org under a CC BY license, with permission from Ishaan Raghunandan, original copyright [2014].

In total, we collected 245 blood samples (153 from *Saguinus fuscicollis*, 92 from *Saguinus imperator*) spread across 129 individuals (83 and 46, respectively) during the study period. Zero infections were confirmed from examination of thin blood smears; however, 10 samples were successfully amplified by nPCR from emperor tamarins (three each from 2012 and 2013, and two each from 2014 and 2015) and one from a saddleback tamarin in 2014 (Table 1). The single infection of a saddleback tamarin only amplified once during preliminary screening for *Plasmodium* following the Duval et al. [42] protocol, and because the *cytb* fragment was of shorter length it was excluded from phylogenetic analysis; however, the sequence was 100% identical to others obtained in this study. The remaining 10 partial *cytb* sequences (1,038bp) were 100% identical to each other and to reference sequences for human isolates of *P*. *malariae* and squirrel monkey (*Saimiri* sp.) isolates of *P*. *brasilianum* (Fig 2); only one sequence per year is included in the phylogeny. These 10 sequences have been deposited in GenBank (Accessions KY709297– KY709306).

**Table 1:** Nested-PCR screening results.

Individuals sampled	Prevalence (*S*. *imperator* only)	Incidence (*S*. *imperator* only)	Animal ID	Species	Sex	Age class	Group ID (size)	Sample Collection Date
*S*.*fuscicollis* 35 *S*. *imperator* 21	0.14	NA	81	*S*. *imperator*	M	Adult	9 (6)	6/13/12
			32	*S*. *imperator*	M	Adult	9 (6)	6/13/12
			89	*S*. *imperator*	F	Adult	15 (3)	6/18/12
*S*. *fuscicollis* 45 *S*. *imperator* 24	0.13	0.33	34*	*S*. *imperator*	F	Sub-adult	9 (7)	7/10/13
			32	*S*. *imperator*	M	Adult	9 (7)	7/10/13
			36**	*S*. *imperator*	F	Adult	9 (7)	7/10/13
*S*.*fuscicollis* 36 *S*. *imperator* 21	0.10	0.0	140	*S*. *fuscicollis*	M	Adult	13 (4)	6/27/14
			32	*S*. *imperator*	M	Adult	9 (6)	7/6/14
			36	*S*. *imperator*	F	Adult	9 (6)	7/6/14
*S*.*fuscicollis* 37 *S*. *imperator* 26	0.08	0.0	32	*S*. *imperator*	M	Adult	9 (8)	6/27/15
			36	*S*. *imperator*	F	Adult	9 (8)	7/3/15

*Infection was not detected in this individual in 2014.

**This individual is natal to this group, born in 2011. An infection was not detected in 2012 as a sub-adult.

Prevalence of infection among emperor tamarins was 0.14 in 2012 (n = 21), 0.13 in 2013 (n = 24), and 0.10 in 2014 (n = 21), and 0.08 in 2015 (n = 26) with an average across years of 0.11+/-0.03 (Table 1). While prevalence remained relatively stable across years, incidence decreased from 0.33 in 2013 to 0 in 2014 and 2015. Prevalence was maintained by 1 adult male that remained infected across the entire study period, and 1 adult female, born in this same group in 2011, that acquired and maintained an infection from 2013 to 2015 (see Table 1). Two other emperor tamarin individuals (an adult male and female from 2012) were found infected in the only years they were sampled. An infection in one sub-adult female from 2013 could not be detected in 2014 or 2015. Although this study assessed 7 emperor tamarin groups, 4 of the 5 emperor tamarin infections belonged to the same group. The infected saddleback tamarin was an adult male from 2014 whose home range partially overlapped with the infected emperor tamarin group and also included parts of the basecamp at the field site. An infection was not detected from this individual in 2015.

## Discussion

A handful of *Plasmodium* species other than *P*. *malariae/brasilianum* can infect both human and nonhuman primates. However, in many cases, there is still limited evidence that non-human primates are a reservoir of human malaria. A clear example of zoonotic malaria is *Plasmodium knowlesi*, a simian parasite in southeast Asia that has been repeatedly found in humans [48,49]. This parasite appears to have independently infected humans in many areas of Southeast Asia [49–51]. In addition, *Plasmodium cynomologi* parasitizes Asian macaques and has at least one documented case in humans [52]. Beyond these two cases, other studies have detected human malarias in non-human primates but the epidemiological and genetic data are still insufficient to implicate non-human primates as reservoirs for human malaria. The human parasite *P*. *vivax* is suspected of circulating in a subset of west African apes that are positive for the Duffy blood group antigen molecule [4]. The normal hosts for *Plasmodium simium* are large New World monkeys (Atelidae), but there is at least one case of a human infection [53]. *P*. *falciparum*, the most virulent human *Plasmodium*, is sometimes detected in New World monkeys (8 species over 5 genera) [5] but there is no evidence indicating that such non-human primates act as malaria reservoirs.

Although host switches are common in non-human primates, not all host switches indicate the presence of a zoonosis [43,54]. As an example, *P*. *falciparum* has been found in apes, particularly chimpanzees [20,55]. However, such infections had a human origin because they were all resistant to commonly used antimalarial drugs [43]. Thus, it was shown that apes could acquire the parasite from humans; however, whether there could be human infections from a non-human primate host (a true zoonosis) requires additional evidence beyond the detection of identical parasites. In particular, evidence of active gene-flow and the presence of competent vectors that can infect humans from a non-human primate are missing. A case in which zoonoses have been clearly established by these criteria is *P*. *knowlesi* from isolated macaque (*Macaca* spp.) populations in Borneo and Peninsular Malaysia [51]. A first step in the case of *P*. *malariae/brasilianum*, however, is to better characterize its host range throughout South America.

*P*. *brasilianum* has been screened for in *S*. *fuscicollis* on two separate occasions between 1995 and 2013 (n = 19 and 6, respectively) in Brazil using only microscopy [9,22], and only once in *S*. *imperator* (n = 2) [56], with zero reported infections for both species. Here we confirm for the first time that these two species are susceptible to *P*. *brasilianum/malariae*. Like past studies of *Plasmodium* from other simian hosts in South and Central America, *P*. *brasilianum* was genetically identical to *P*. *malariae* using *cytb*, reinforcing that they are likely to be a single organism [14]. That we were only able to amplify a single saddleback tamarin infection in a single assay is likely caused by poor sample quality, extremely low parasitemia, or both. As the sample was physically isolated from any other surrounding positives, it would represent a very improbable instance of contamination during laboratory analyses.

Importantly, our data suggest that chronic infections of *P*. *brasilianum* occur in the wild, consistent with the low, stable prevalence in emperor tamarins despite decreasing incidence of new infection (Table 1). If true, this provides evidence that Callitrichidae might act as reservoirs for human zoonotic malaria; however further investigation should take place to show that a complete parasite lifecyle is taking place, such as the presence of intraerythrocytic development. Given the low diversity of *Plasmodium* parasites that infect primates in this area, the possibility of reinfections should not be ruled out. Our findings also suggest that these non-human primates may naturally clear infection of *P*. *brasilianum*; however, it will be necessary to differentiate a natural clearance from a sub-microscopic infection with a parasitemia that is below PCR detection thresholds. Since infections appear to be clustered in our study population, additional years of data will allow us to track the rate of transmission to new group members (for example, offspring within infected groups) and to new groups. This also opens possibilities for measuring individual health parameters before and after the onset of *P*. *brasilianum* infections and whether there exist associations with other natural parasites [21].

The parasite prevalence we observed for emperor tamarins was in the same range that has been published from other wild Neotropical primate populations. Although prevalence was too low to analyze variation between different demographic groups, we observed that 4 of 5 infections occurred in a single group (out of 7). Previous studies on *Plasmodium* from Neotropical primates make little mention of how parasites are distributed within host populations, but potentially uneven distributions would be an important factor for assessing disease risk [51], particularly if some of those non-human primates share competent vectors with humans. Although the available data are limited, there are several explanations for the observed pattern of clustered infections. First, the mosquito vector might show preference for certain vertebrate hosts. This hypothesis requires additional data, including some evidence of population structure in the parasite that is linked to specific hosts. Indeed, *Plasmodium inui*, also a quartan parasite, does not show host population structure in Borneo [57] but *P*. *knowlesi* does in Malaysia [51]. This effect could even be exaggerated by host behavior, if, for example, the infected group utilizes unusually open sleep site locations. Nunn and Heyman [58] found preliminary support for the hypothesis that primates that sleep in closed microhabitats experience lower prevalence of *Plasmodium* infection. Emperor tamarins generally sleep in thick tangles of branches and vines, and sometimes tree holes, and although this is an unlikely explanation, it will be worth ruling out in future years. Second, there could be additional hosts within the home range of the infected group of *S*. *imperator* that might increase the parasite encounter rate. The latter scenario is not unlikely, as there are 9 other nonhuman primate species present that could host *Plasmodium*, including members of the Atelidae, Cebidae, Pithecidae, and Aotidae, as well as a small but dynamic population of human researchers. However, all of the nonhuman primate species occur concurrently throughout the study area, and thus may not explain clustering within one species and one social group. Regarding the risk from infected humans at EBLA, the home range of this group does not overlap with the stations basecamp. Of two saddleback and two emperor tamarin groups with home ranges that do intersect with the basecamp, only the sole saddleback tamarin that appeared infected for a single year is a member of these groups. That these groups closest to basecamp, which are the most well-sampled and exposed to the highest degree of proximity with researchers, accounted for a single *Plasmodium* infection from one year, suggests that transmission from human to nonhuman primates is not the source of *P*. *brasilianum* infection at EBLA. However, none of the resident staff or researchers were tested for infection even though asymptomatic human carriers of *P*. *malariae* have been reported [59,60], and in general, greater efforts to detect *P*. *malariae* in human populations that are in contact with non-human primates infected with *P*. *brasilianum* are needed to fully assess whether there are zoonotic infections. Finally, there could be differences in host susceptibility or simply very low parasitemia below the detection of the PCR implemented in this study. As we have only sampled tamarin groups that occur within an approximate 200-hectare area, it would be worthwhile to expand the study area to see if other clusters are present.
